# A long-run convergence analysis of aerosol precursors, reactive gases, and aerosols in the BRICS and Indonesia: is a global emissions abatement agenda supported?

**DOI:** 10.1007/s11356-022-22988-9

**Published:** 2022-09-29

**Authors:** Diego Romero-Ávila, Tolga Omay

**Affiliations:** 1 Department of Economics, Universidad Pablo de Olavide, Ctra de Utrera, Km. 1, Sevilla, Spain; 2 Atılım University, Kızılcaşar Mahallesi, 06830 İncek Gölbaşı, Ankara, Turkey

**Keywords:** GHG emission convergence, Nonlinearities, Unit root, Time-dependence, State-dependence, Global Emissions Abatement Agenda, 2030 Agenda for Sustainable Development, C24, C33, Q50, Q53, Q54, Q58

## Abstract

This article examines the hypothesis of deterministic emissions convergence for a panel of the BRICS and Indonesia to advanced countries’ emissions levels as well as to Sweden (which is a country that has clearly gone through decoupling) using a novel dataset with ten series of annual estimates of anthropogenic emissions comprising aerosols, aerosol precursor and reactive compounds, and carbon dioxide from 1820 to 2018. For that purpose, we employ four novel panel unit root tests allowing for several forms of time-dependent and state-dependent nonlinearity. The evidence supports deterministic convergence following a linear process for carbon dioxide, whereas the adjustment is asymmetric and nonlinear for carbon monoxide. Methane and nitrogen oxides exhibit logistic smooth transition converging dynamics. In contrast, black carbon, ammonia, nitrous oxide, non-methane volatile organic compounds, organic carbon, and sulfur dioxide emissions diverge. These results have implications for the abatement of greenhouse gases emissions at the global level, given the high share of emissions of the BRICS.

**Supplementary Information:**

The online version contains supplementary material available at 10.1007/s11356-022-22988-9.

## Introduction

Hoesly et al. (2018) point out that “anthropogenic emissions of reactive gases, aerosols, and aerosol precursor compounds have substantially changed atmospheric composition and associated fluxes from land and ocean surfaces.” The associated atmospheric chemical reactions have given rise to the ozone hole, global warming, and climate change, which have altered ecosystems and worsened human health.

The ozone-depleting phenomenon derives from photochemical reactions of chlorine compounds caused by the combination of polar stratospheric clouds made up of sulphuric acid, nitric acid and water at very low temperatures, and sunlight (Hidy 2001). Sulfuric acid stemming from sulfur dioxide emissions in contact with air and water gives rise to acid rain, which harms ecosystems, human health, plants’ life, and the economy by affecting crops (Solarin and Tiwari 2020).^1^ Like sulfur dioxide, nitrogen oxides mostly emerge from fossil fuel combustion at high temperatures and constitute a precursor to photochemical smog and acid rain. Nitrogen oxides also contribute to ground-level ozone accumulation^2^ as well as to the phenomenon called eutrophication in coastal waters—that hampers biodiversity and brings toxicity—hence harming both terrestrial and aquatic ecosystems. They also cause respiratory and cardiovascular illnesses in humans, thereby impairing plant growth and crop yields (Solarin et al. 2021). Ammonia is a gas which, despite not being considered directly a greenhouse gas (GHG), can act as a precursor to nitrous oxide, which is a powerful GHG.^3^ Like nitrogen oxides, ammonia contributes to acid rain and eutrophication of coastal waters and rivers, damaging aquatic and land ecosystems, crop yields, and human health (Solarin et al. 2022a).

After carbon dioxide, the next contributor to GHG emissions is methane with 16% of total emissions. As pointed out by the US Environmental Protection Agency (2021), pound for pound, comparatively the global warming potential and global temperature contribution of methane is 25 times higher than that of carbon dioxide emissions over a century-long period. This is because methane gas is a potent heat trapper and has a longer atmosphere lifetime than carbon dioxide (Solarin et al. 2022b).^4^ Methane is a chemically reactive gas which, when oxidating, brings a rise in carbon emissions and stratospheric water vapor, thereby distorting the amount of other compounds such as tropospheric ozone and hydroxyl (Wuebbles and Tamaresis 1993; Solarin et al. 2022b). As pointed out by Solarin and Gil-Alana (2021), despite not directly harming crop yields or human health, the ozone generated with the oxidation of methane is responsible for respiratory morbidity and premature mortality.

Incomplete fossil fuel combustion releases carbon monoxide, volatile organic compounds, as well as particles of organic carbon and black carbon. The latter is particularly pervasive because it contributes to global warming by converting incoming solar radiation to heat. If deposited on ice and snow, it reduces surface albedo (i.e., the ability to reflect sunlight). Hence, it heats the surface, thus causing the ice melting of the Arctic and glaciated regions (Climate and Clean Air Coalition 2022). In addition, Hidy (2001) argues that atmospheric chemical reactions are responsible for many of the existing aerosol precursor particles. The oxidation of reactive gases such as sulfur dioxide, nitrogen oxides, and non-methane volatile organic compounds (benzene, xylene, propane and butane) leads to the production of small particles in the atmosphere. Fossil fuel combustion constitutes the main source of these compounds. The formed sulfate aerosols enter the clouds, which leads to reflecting more sunlight. This gives rise to the cooling phenomenon in the atmosphere, which is opposite to global warming by greenhouse gases, though mainly acting regionally near industrialized areas (NASA 2017).^5^

Environmental authorities worldwide have reacted to tackle these climate challenges through the 2030 Agenda for Sustainable Development endorsed globally by the United Nations Environmental Programme and the adoption of the Paris Agreement. More specifically, the 2030 Agenda entails 17 goals, of which Sustainable Development Goal 13 is key to achieve the 2030 Agenda and the Paris Agreement on climate change. This goal seeks to combat climate change via the provision of sufficient financial flows, enhanced technology and human and institutional capacity building as well as raising public awareness. Likewise, the endorsement of the 2015 Paris Agreement tackles climate change by keeping the rise in global temperature this century below two degrees Celsius above pre-industrial levels.

Given the policy implications of the above climate challenges, this article examines the hypothesis of deterministic convergence among a panel of the BRICS and Indonesia for ten series of annual estimates of anthropogenic emissions that include two carbonaceous aerosols (black carbon and organic carbon), aerosol precursor and reactive compounds (carbon monoxide, nitrous oxide, nitrogen oxides, sulfur dioxide, ammonia, methane, and non-methane volatile organic compounds), and carbon dioxide between 1820 and 2018. This is very relevant for climate change policies since until now countries have only focused on curbing carbon dioxide emissions, thus ignoring other pollutants whose emissions remain high (Stern 2014). Besides, the focus on the emissions convergence of the BRICS and Indonesia to advanced countries’ levels is because they constitute large emitters (particularly China and India) and their failure to converge would compromise the achievement of the global environmental policy agenda. As a matter of fact, according to Boden et al. (2017), in 2014 China was the main global emitter of carbon dioxide from fossil fuel combustion and cement manufacturing and gas flaring, amounting to 30% of total emissions. The second main emitter was the USA with 15%, followed by the EU-28 with 9%, India with 7%, the Russian Federation with 5%, and Japan with 4%.

In the empirical analysis, we employ four novel panel unit root tests that allow for several forms of time-dependent and state-dependent nonlinearity. Indeed, this paper provides a novel contribution in that no previous study has analyzed the emissions convergence of the BRICS and Indonesia to the levels of the developed world for such a large number of pollutants over two centuries by means of several nonlinear panel unit root tests allowing for such rich nonlinear dynamics. Our findings support deterministic convergence following a linear process for carbon dioxide, whereas the adjustment is asymmetric and nonlinear for carbon monoxide. Methane and nitrogen oxides exhibit logistic smooth transition converging dynamics. In contrast, black carbon, ammonia, nitrous oxide, non-methane volatile organic compounds, organic carbon and sulfur dioxide emissions diverge. These results have implications for the abatement of greenhouse gases emissions at the global level, given the high share of emissions of the BRICS. More specifically, the fact that these major emitters converge to emissions levels in the developed world for the main greenhouse gas, carbon dioxide, along with three other compounds supports the global emissions abatement agenda given by the 2030 Agenda and the Paris Agreement. Notwithstanding, as explained in the Policy Implications section, the BRICS and Indonesia should make more efforts to curb emissions in the six compounds that fail to converge to advanced countries’ emissions levels.

The remainder of the paper is organized as follows. The “Short notes on BRICS and Indonesian climate policy” section provides some notes on the BRICS regarding their emissions control policy efforts. The “Literature review” section reviews the literature on pollutants emissions convergence using unit root testing. The “Brief econometric notes” section briefly describes the panel nonlinear unit root tests used in the empirical analysis, leaving the econometric details to the unpublished appendix. The “Data and empirical strategy” section presents the data, empirical strategy and current status of pollutants’ emissions. The “Empirical results” section reports the findings, and the “Conclusion and policy implications” section provides some policy implications and concludes.

## Short notes on BRICS and Indonesian climate policy^6^

The BRICS possess nearly 30% of the world’s land mass and are inhabited by over 40% of the world’s population. According to Tian et al. (2020), using data from the World Development Indicators of the World Bank, total aggregate output (in 2011 international dollars) has risen to 17.6% in 1995 to 32.5% in 2018, with 2050 projections pointing to China, India, Russia, and Brazil as the first, third, fifth, and sixth largest economies in the world. Interestingly, the BRICS tend to specialize in different sectors depending on their strengths. China specializes in manufacturing, India in services, Brazil and South Africa in agriculture, and Russia in fossil fuel energy sources (Azevedo et al. 2018). It is also remarkable that, with the exception perhaps of Russia, the BRICS exhibit particular vulnerability to the risks and impacts of global-warming-induced climate change (BRICS 2015).

In 2009 the BASIC coalition was formed as a negotiation group by China, the country with the highest emissions, and India, Brazil and South Africa with strong emissions growth. The coalition led the negotiation with the USA to approve the Copenhagen Accord in the Conference of the Parties (COP) 15. These countries defend the position that the main cost of climate change mitigation policies should continue to be incurred by the industrialized world since their per capita emissions are still well above those of emerging countries. As such, they reject binding obligations of emissions reductions and defend the primacy of their economic development under the principle of common but differentiated responsibility and respective capabilities. Besides, they seek to obtain green development finance from the developed world to conduct adaption and mitigation actions (Falkner 2016; Downie and Williams 2018).

In the Paris Agreement BASIC countries worked and negotiated together, refusing to accept national binding emissions commitments (Michaelowa and Michaelowa 2015, 2020). In contrast, Russia^7^ did not support any (even non-binding) substantial mitigation effort, which could compromise Russian fossil fuel export revenues. Furthermore, Downie and Williams (2018) point out that India and China, due to their huge energy needs, are interested in reducing their dependence on imported fossil fuels, whereas Russia and to a less extent Brazil—as large oil and gas exporters—are interested in expanding exports and increasing energy prices. Hence, these differing interests and incentives make it more likely that the BASIC coalition will continue to act as a negotiation group in climate change issues over a BRICS coalition. Let us now briefly present their main position and policy actions against GHG emissions, drawing in many instances from Gladun and Ahsan (2016).

### Russian Federation

For Russia, which has vast land areas far north of the Equator, warmer temperatures could transform frozen areas to arable and mineral-exploitable lands. In addition, unlike other geographical regions, only a small proportion of the Russian population shows vulnerability to climate change (Gladun and Ahsan 2016). On top of that, Russia is a major fossil fuel exporter of natural gas and oil. All this explains the scepticism of Russian authorities to legislate on climate issues. The “non-binding” Climate Doctrine approved in 2009—containing guidelines for future climate policy—has not translated into specific legislation targeting emissions cuts. Russian authorities feel that (1) the introduction of restrictive emission mitigation measures would impede their economic development and (2) their participation in international climate agreements would put at risk their fossil fuel export revenues.

Russian Energy Strategy targets the hydrocarbon, nuclear and hydropower sectors, while the renewable energy sector is almost neglected (Gladun and Ahsan 2016). The Intended Nationally Determined Contribution (INDC) within the 2015 Paris Agreement is to reduce GHGs emissions by 25–30% below 1990 levels by 2030. Given the economic decline of the past decades, this voluntary pledge does not represent any significant effort to cut emissions.^8^

### India

Unlike Russia, India is a country highly vulnerable to climate change hazards, in which traditional biomass is the main source of energy in rural areas (Michaelowa and Michaelowa 2020). Under the Kyoto Protocol, India participated in more than 1500 registered Clean Development Mechanism projects, receiving a large flow of financial resources from the developed world to combat climate change (Gladun and Ahsan 2016).

In addition, India has been very active in promoting energy legislation measures, so that the reduction of emissions does not come at the expense of economic development. The National Mission on Enhanced Energy Efficiency aims at enhancing energy-efficient technologies by fostering investments in the energy sector. The Energy Conservation Building Code provides a guide to the construction sector for energy-saving buildings. India’s National Electricity Policy of 2005 actively promotes the development of renewable energy sources such as solar and wind energies. It also promotes nuclear power, public transportation and energy pricing reform toward greater competition in the energy sector. The INDC of India implies a reduction of emissions intensity by 33%-35% below 2005 levels by 2030, a share of renewables in installed electricity power capacity of 40% by 2030, and expanding reforestation to create carbon sinks (Gladun and Ahsan 2016).

### China

Like India, China is being very active in legislating energy conservation measures. China’s National Climate Change Program released in 2007 targets the reduction of energy intensity. For that purpose, the Energy Conservation Law aims at improving energy efficiency to slow down the growth of energy demand, and the Renewable Energy Law promotes the use of renewable energy sources like solar, wind and hydropower. In addition, the Program seeks to expand nuclear power and natural gas. These, along with renewable energy sources, should displace the highly polluting combustion of coal.^9^ The Program also aims at closing inefficient industrial plants, improving building construction standards and creating carbon sinks through reforestation (Gladun and Ahsan 2016).

China has extensively taken part in Clean Development Mechanism projects, amounting to over 40% of the global emission credits granted by this mechanism. China’s INDC under the Paris Agreement implies a reduction of emissions intensity by 60-65% below 2005 by 2030, reaching the CO_2_ emissions peak that year. It also seeks to raise the share of non-fossil fuel energy carriers of the total primary energy supply to 20% by 2030 and create carbon sinks by reforestation. As pointed out by Gladun and Ahsan (2016), the INDC carbon intensity target appears insufficient to control the level of emissions by 2030, given the rapid growth of the Chinese economy.

### Brazil

Brazil’s contribution to global GHG emissions is about 3%, most of which stems from deforestation. Unlike other BRICS, the energy sector, which is based on hydropower, is not driving GHG emissions. Nonetheless, Brazil’s oil and gas are gaining momentum and GHG emissions from fossil fuel combustion are expected to rise significantly. The National Plan on Climate Change, which was updated in December 2014, seeks to reduce emissions by 36.1–38.9% by 2020 relative to 2005. Four key areas are targeted: deforestation, agriculture and livestock, energy sector, and steel sector. The Brazilian government aims to raise energy efficiency and renewable energy sources, increase the already high biofuels use in the transport sector and in agriculture, reduce deforestation, and raise investment in adaptation and mitigation as well as in low-carbon technologies.

Brazil’s INDC implies a reduction in GHG emissions by 37% in 2025 and 43% in 2030 below 2005 levels. Reaching a 45% share of renewables in the total energy supply by 2030 and the receipt of external financial support for adaptation technologies will be key to achieve the emissions reduction targets (Gladun and Ahsan 2016).

### South Africa

Like China and India, South Africa is a large coal mining producer, which is the main energy source for power generation (Downie and Williams 2018). As a developing country, it needs to make compatible economic development with realistic emissions reduction commitments. South Africa launched an ambitious program that seeks to produce 300 GW of electricity for the African continent by 2030. This Africa Renewable Energy Initiative pursues sustainable development by increasing the use of renewable energy sources and developing low-carbon technologies. This is key to achieve universal access to clean and affordable energy, in line with SDG7 of the 2030 Agenda.

The 2011 National Development Plan pursues the idea of a green economy fostered by a competitive and efficient energy sector, in which renewables play a key role. Even though some traditional coal-fired power plants have been closed, more efforts should be made since coal combustion is still prevalent in power generation (Guo et al. 2021). More recently, South African authorities approved the Integrated Resource Plan in October 2019. This plan foresees a large shift in energy generation from coal to renewables. Thus far, the full implementation of the plan remains uncertain. In addition, the government introduced a carbon tax in February 2019. It is applied to emissions stemming from fossil fuel combustion, industrial processes and coal mining, among others. Since the first phase of the plan entails tax exemptions for up to 95% of emissions until 2022, it has not yet had a significant impact (Climate Action Tracker 2021).

South Africa’s INDC aims to keep GHG emissions within 398–614 metric tons of CO_2_ equivalent (MtCO2e) by 2025–2030, respectively (Dong et al. 2017). This target has been updated in 2021 to an absolute emissions level in the range of 350–420 MtCO2e for 2030 (Climate Action Tracker 2021).

### Indonesia^10^

Indonesia is expanding coal use in power generation until 2027, which is expected to represent 64% of electricity generation by 2030. In addition, rapid deforestation caused by expanding palm-oil plantations has led to a share of the country’s total emissions of almost 50% over the last 20 years (Climate Action Tracker 2021). With the introduction of the Indonesian Sustainable Palm Oil Scheme, which requires all palm-oil plantations to be certified, the government seeks to stop deforestation and social conflicts.^11^ The implementation of the One Map Policy should closely monitor forest areas to control deforestation practices.

Within the General Plan of National Energy published in 2007, the new electricity sector plan is not sufficiently ambitious to envisage a shift from coal-fired power generation to renewable energy sources, despite the fact the country has potential for solar power, hydropower, wind power, bioenergy, geothermal, and wave power (Climate Action Tracker 2021). Indonesian authorities are supporting the use of biofuels and electric vehicles in the transport sector. It is mandated that biofuels^12^ represent at least 20% of fuel consumption since 2016 and prior to 2020, increasing this share to 30% by 2020. The Electric Vehicles Development Plan targets over 2,000 four-wheel electric vehicles, 700,000 hybrids, and over two million of two-wheel electric vehicles by 2025, increasing these numbers to four, eight and 13 million by 2050 (Climate Action Tracker 2021).

Indonesia’s INDC implies an unconditional 29% reduction in total emissions below a business-as-usual scenario by 2030, while a conditional 41% reduction in total emissions below a business-as-usual scenario by 2030. The 2021 update confirms the initial 2030 targets.

## Literature review

For the sake of conserving space, this literature review focuses only on those studies employing univariate or panel unit root and stationarity statistics to examine time-series definitions of emissions convergence in global samples or small samples of countries, paying particular attention to the results for industrialized and emerging countries.^13^

### Convergence in CO_2_ emissions

Aldy (2006) examines stochastic convergence with univariate generalized least squares (GLS) augmented Dickey and Fuller (1979) (ADF) tests for per capita CO_2_ emissions of 23 OECD countries and a global sample of 88 countries over the period 1960–2000. He finds mixed evidence for the Organization for Economic Co-operation and Development (OECD) sample while divergence for the full sample. Researchers have taken several avenues to try to overcome the caveats associated with the ADF statistic leading to low statistical power in finite samples (Campbell and Perron 1991), as well as in the event of not incorporating sharp structural breaks, thresholds and smooth nonlinearities (Perron 1989; Kapetanios et al. 2003).

The first avenue is raising statistical power by exploiting the panel dimension of the datasets. Employing the Im et al. (2003) panel unit root test for per capita CO_2_ emissions in 21 OECD countries over the period 1960–1997, Strazicich and List (2003) find evidence of stochastic convergence. Westerlund and Basher (2008) investigate stochastic convergence in per capita CO_2_ emissions through three panel unit root tests with a common factor representation for a sample of 16 OECD and 12 developing countries over the period 1870–2002. Strong evidence of convergence is found, with an unbiased half-life estimate of 6 years. Applying seemingly unrelated (SUR) ADF tests to per capita CO_2_ emissions of 21 OECD countries over the period 1960–2000, Lee and Chang (2008) find evidence of stochastic convergence in only seven countries. Employing a battery of univariate and panel stationarity and unit root tests on per capita CO_2_ emissions of 21 OECD countries over the period 1950–2002, Barassi et al. (2008) find no evidence of stochastic convergence. Along similar lines, Karakaya et al. (2019) apply univariate and panel cross-sectionally augmented ADF tests to per capita CO_2_ emissions in 16 OECD countries over the period 1960–2013. The evidence does not support stochastic convergence.

The second avenue is to incorporate sharp structural breaks in the univariate and panel unit root statistics testing emissions convergence. Using univariate Lagrange multiplier (LM) unit root tests allowing for two structural breaks, Chang and Lee et al. (2008) find evidence of stochastic convergence in per capita CO_2_ emissions for a sample of 21 OECD countries over the period 1960–2000. Likewise, Lee et al. (2008) employ univariate unit root tests with structural breaks for analyzing per capita CO_2_ in 21 OECD countries between 1960 and 2000. The evidence favors stochastic convergence in 13 countries. Using two-break LM and residual-augmented least-squares-regression (RALS)-LM unit root tests, Ozcan and Gultekin (2016) find evidence of stochastic convergence in per capita CO_2_ in most of the 28 OECD countries studied over the period 1960–2013 when structural breaks are incorporated. Employing RALS-LM unit root tests with up to two breaks for examining per capita CO_2_ emissions of 30 OECD countries over the period 1900–2014, Awaworyi-Churchill et al. (2018) find stochastic convergence during the whole period, and more pronounced over the postwar period. Using the same statistics, Awaworyi-Churchill et al. (2020b) examine per capita CO_2_ emissions for a sample of 17 emerging countries between 1921 and 2014. They find convergence in 11 countries including China, Indonesia, and Russia, while six countries (including Brazil and India) fail to converge. Using LM and RALS-LM unit root tests with breaks, Solarin (2019) finds stochastic convergence in per capita CO_2_ emissions among 22 of the 27 OECD countries studied over 1961–2013.

Among the studies incorporating structural breaks in panel statistics, we find the following. Focusing on per capita CO_2_ emissions of 23 OECD countries between 1960 and 2002, Romero-Avila (2008) employs the panel stationarity test with multiple breaks of Carrion-i-Silvestre et al. (2005)—CBL hereafter. He provides strong evidence of both stochastic and deterministic convergence. Likewise, Lee and Chang (2009) apply the same test to per capita CO_2_ of 21 OECD countries over 1950–2002, finding evidence of stochastic convergence.

The third path incorporates nonlinearities in the univariate and panel statistics employed. Using the nonlinear unit root test of Kapetanios et al. (2003), Camarero et al. (2011) find no evidence of stochastic convergence in per capita CO_2_ emissions among 22 OECD countries between 1950 and 2006. Presno et al. (2018) employ univariate stationarity tests allowing for quadratic trends with smooth transitions to analyze per capita CO_2_ among 28 OECD countries over 1901–2009. Their evidence favors stochastic convergence. Yavuz and Yilanci (2013) employ a threshold autoregressive (TAR) panel unit root test to study per capita CO_2_ for the G7 countries over 1960–2005. Data are split into two regimes, with convergence in the first regime, while divergence in the second partly caused by the oil shocks of the 1970s. Using the panel stationarity test with multiple breaks of CBL and an extended version with a Fourier function, Erdogan and Acaravci (2019) analyze per capita CO_2_ emissions among 28 OECD countries over 1960–2014. Evidence favors convergence with the CBL test, but the evidence is mixed with the Fourier extension. Using the same panel statistics, Cai and Wu (2019) find evidence of stochastic convergence in per capita CO_2_ for 21 OECD and 19 emerging economies over 1960–2014 with the panel test of CBL, and only for 11 OECD and 10 emerging countries (including China, India, and Indonesia) with the Fourier extended version. Using a Fourier-based wavelet ADF test with smooth shifts, Erdogan and Solarin (2021) investigate stochastic convergence on per capita CO_2_ emissions among 151 counties of which 53 are high-income countries. The evidence favors convergence in 35 high-income countries, 27 upper-middle-income countries, 30 lower-middle-income countries, and 13 low-income countries.

The fourth avenue is the use of fractional integration techniques to measure more precisely the degree of persistence of emissions converging dynamics and the pairwise approach of Pesaran (2007). Using the local Whittle estimator and fractional integration tests, Barassi et al. (2011) find evidence of fractional integration supportive of slow convergence in per capita CO_2_ emissions in 13 out of 18 OECD countries between 1870 and 2004. Employing fractional integration tests with structural breaks, Barassi et al. (2018) find stochastic convergence in per capita CO_2_ in only 30 to 40% of the 28 OECD countries studied over 1950–2013. Allowing for structural change via Chebyshev polynomials and nonlinearities via the multivariate adaptive regressions splines model, Sephton (2020) finds evidence of convergence in per capita CO_2_ in nearly all the 28 OECD countries series over the period 1950-2014.

In all, the evidence points to convergence for specific groups of countries sharing a comparable development level (particularly industrialized countries), while the evidence favors divergence for global samples or samples involving countries with differing degrees of development.

### Convergence in other compounds than CO_2_

The study of convergence of other compounds than CO_2_ has received little attention in the literature. There are some exceptions that we next point out. Focusing on stochastic convergence in CH_4_ emissions among 37 OECD countries over the period 1781–2019, Solarin et al. (2022a) employ a Fourier-based wavelet unit root statistic to show overwhelming evidence in favor of divergence in methane emissions. Employing the pairwise approach of Pesaran (2007) and the panel stationarity test with multiple breaks of CBL, El-Montasser et al. (2015) find no evidence of stochastic convergence in CO_2_, CH_4_, N_2_O, petrofluorocarbons (PFCs), hydrofluorocarbons (HFCs), and sulfur hexafluoride (SF_6_) among the G7 countries over 1990–2011.

Applying a panel Fourier threshold unit root test to aggregate and sectoral NO_x_ in G7 countries between 1750 and 2019, Solarin et al. (2021) find evidence of full convergence for agriculture, energy production, and transport sectors, while partial convergence in the aggregate and remaining sectors. Using a Fourier-augmented wavelet unit root test, Solarin et al. (2022b) analyze stochastic convergence in NH_3_ emissions among 37 OECD countries over the past two centuries. They focus on ammonia emissions at the aggregate level, sectoral level and by fuel source, with most evidence pointing to divergence. Solarin and Tiwari (2020) study stochastic and deterministic convergence in per capita SO_2_ among 32 OECD countries between 1850 and 2000 using the panel stationarity test with breaks of CBL, and a panel stationarity test with a common factor and a Fourier function. The evidence favors both notions of convergence with the CBL test, while the evidence is mixed with the Fourier-based panel test. Nourry (2009) employs the pairwise approach of Pesaran (2007) to examine stochastic convergence in per capita CO_2_ and SO_2_ emissions among 127 and 81 countries, respectively, over 1950–2003, failing to find support of convergence even among OECD countries.

At the disaggregate level, there are five studies on SO_2_ emissions convergence: two for China and three for the USA. Zhang et al. (2020) employ a Fourier quantile unit root test to investigate stochastic convergence in per capita SO_2_ emissions across 74 cities of China between December 2014 and June 2019. Asymmetric nonlinear convergent dynamics are found in 72 out of 74 cities. Employing dynamic panel data estimators, Hao et al. (2015) find evidence of absolute and conditional convergence in per capita SO_2_ emissions across 113 Chinese cities over the 2002–2012 period. Convergence is observed within the whole nation, as well as in the eastern, western, and central regions of China.

Payne et al. (2014) employ the RALS-LM unit root test with breaks to examine stochastic convergence in per capita SO_2_ emissions across the US states over the period 1900–1998, finding evidence of convergence for all states but five (for which the unit root null could not be rejected). Using the Perron and Vogelsang (1992) innovation-outlier trend-break model, List (1999) finds evidence of stochastic convergence in per capita SO_2_ and NO_x_ emissions across 10 Environmental Protection Agency (EPA) regions over the 1929–1994 period. Along similar lines, Bulte et al. (2007) use the Lee and Strazicich (2003) unit root test with breaks to study stochastic convergence in per capita SO_2_ and NO_x_ emissions across the US states over the period 1929–1999. Evidence in favor of convergence is particularly apparent during the period of federal pollution control (1970–1999) resulting from the introduction of the Clean Air Act in 1970 (US Environmental Protection Agency 2012).

Our empirical analysis clearly differs from the aforementioned studies in that we investigate a much larger number of compounds using nonlinear panel unit root statistics accounting for nonlinear dynamics that have not been employed so far in the literature. Allowing for such rich nonlinear dynamics in the emissions convergence process will enable us to characterize the data generation process (DGP) of each compound.

## Brief econometric notes

A growing literature has relaxed the linearity assumption and developed statistics that test linear nonstationarity against the alternative of nonlinear stationarity. The two main sources of nonlinearity are state-dependent nonlinearity (i.e., nonlinearity in the speed of mean reversion) and time-dependent (structural breaks) nonlinearity (i.e., nonlinearity in the deterministic components). The former type includes Kapetanios et al. (2003)—KSS hereafter—and Sollis (2009). KSS employ the symmetric exponential smooth transition autoregressive model. Sollis (2009) considers the asymmetric exponential smooth transition autoregressive model that allow the speed of convergence to differ across regimes. The latter type includes Leybourne et al. (1998)—LNV hereafter—and Enders and Lee (2012)—EL hereafter—who develop nonlinear structural-break unit-root tests that allow for a single permanent break through a logistic smooth transition function and multiple smooth changes through a flexible Fourier function, respectively. More recently, four respective panel versions of these univariate unit root tests have been developed, which are the ones we employ: the tests of Uçar and Omay (2009)—UO hereafter—Emirmahmutoglu and Omay (2014)—EO hereafter—Omay et al. (2018b)—OHS hereafter—and Omay et al. (2021)—OSS hereafter.

There are some motivations for the existence of nonlinearities in emissions convergence. Oil price shocks impact nonlinearly on economic activity. Since a large proportion of emissions stems from economic activity, nonlinearities in the latter will be directly transmitted to emissions (Presno et al. 2018). In addition, asymmetries in the duration of the business cycle phases, mostly deriving from asymmetric energy price shocks, lead to asymmetries in the duration of cyclical phases of emissions (Awaworyi-Churchill et al. 2020a; Zerbo and Darné 2019).

With the following smooth transition specification for the per capita emission series, we explain the main features of the panel tests:

$${y}_{i,t}={\alpha}_i+{\beta}_1{y}_{i,t-1}+{\beta}_2F\left({y}_{i,t-1},{\theta}_i,{c}_i\right)+{\varepsilon}_{i,t}$$

where *F*(∙) is a transition matrix function, *θ*_*i*_ is the speed of transition between regimes, and *c*_*i*_ represents a threshold parameter. The exponential smooth transition autoregressive (ESTAR) model considered in the UO test is:

$$F\left({y}_{i,t-1},{\theta}_i,{c}_i\right)=1-\mathit{\exp}\left[-{\theta}_i{\left({y}_{i,t-1}-{c}_i\right)}^2\right]$$

where *y*_*i*, *t* − 1_ is lagged per capita emissions for country *i* at time *t*. Size nonlinearity implies that the coefficient on per capita emissions gradually changes taking into account whether per capita emissions are close or far away from equilibrium, regardless whether this deviation is positive or negative. Thus, when there is a very large deviation from equilibrium (i.e., (*y*_*i*, *t* − 1_ − *c*_*i*_) →  ± ∞), the coefficient becomes *β*_1_ + *β*_2_. When there is no deviation (i.e., *y*_*i*, *t* − 1_ = *c*_*i*_), the coefficient is *β*_1_. In the case of the asymmetric exponential smooth transition autoregressive (AESTAR) model, EO employ both an exponential and a logistic smooth transition function to capture asymmetric nonlinear mean reversion towards equilibrium across regimes. Thus, unlike the UO test, the EO test permits positive and negative deviations to revert to equilibrium at different convergence speeds.

Concerning the logistic smooth transition (LSTR) model in the OHS test^14^, we have:

$$F\left({\theta}_i,{c}_i\right)=\frac{1}{1+{exp}\left[-{\theta}_i\left(t-{c}_iT\right)\right]}$$

This transition function is continuous, bounded between 0 and 1, and controls the transition from one regime to another, with the state variable being time. The parameter *c*_*i*_ entails the timing of the transition midpoint. The parameter *θ*_*i*_, implies the smoothness of transition. For small values of *θ*_*i*_, *F*(*θ*_*i*_, *c*_*i*_) crosses the interval (0, 1) very slowly, while for large values of *θ*_*i*_, *F*(*θ*_*i*_, *c*_*i*_) changes from 0 to 1 instantaneously at time *t* = *c*_*i*_*T*. According to structural break nonlinearity, if (*t* − *c*_*i*_*T*) →  − ∞, the model exhibits a pre-break mean level, while if (*t* − *c*_*i*_*T*) →  + ∞ the model presents a post-break mean level. With this specification, policy authorities can curb emissions by controlling this long-term smooth stationary upward trend structure.

Univariate EL tests form the basis of the OSS test. They adopt the LM detrending method and a flexible Fourier function form to allow for multiple smooth breaks that could be present over this lengthy period. We use multiple frequencies that give a more precise approximation than cumulative frequency which overfilters the data (see Shahbaz et al. 2019). Sieve bootstrap algorithms are employed in the computation of each of the panel unit root tests to allow for cross-sectional dependencies of unknown form.

## Data and empirical strategy

### Data description and country sample

We use a novel database for ten series of annual estimates of anthropogenic emissions from the Community Emissions Data System (CEDS) for Historical Emissions (Hoesly et al. 2018; O’Rourke et al. 2021).^15^ In addition to the most important GHG—CO_2_ (carbon dioxide)^16^—we have two major carbonaceous aerosols such as BC (black carbon) and OC (organic carbon), and seven series of reactive gases and aerosol precursor compounds such as carbon monoxide (CO), nitrous oxide (N_2_O), nitrogen oxides (NO_x_), sulfur dioxide (SO_2_), ammonia (NH_3_), methane (CH_4_), and non-methane volatile organic compounds (NMVOCs). The unit of measure for each pollutant is thousand metric tonnes (kt), also known as kilotonnes. The data span over the period 1820–2018 for seven of the pollutants, with the exception of CO_2_ emissions that span over the period 1851–2018 and CH_4_ and NO_2_ that span between 1970 and 2018. Our sample of countries includes the five BRICS (Brazil, China, India, Russian Federation, and South Africa) plus Indonesia. The latter is included in the analysis on the basis that this emerging country shares many economic features of the BRICS and has a large population of almost 300 million inhabitants.

As pointed out in the introduction, we focus on emissions convergence of the BRICS and Indonesia to advanced countries’ levels because they constitute large emitters (particularly China and India). Hence, their failure to converge would compromise the achievement of the global environmental policy agenda, both directly and indirectly by discouraging other emerging economies and the developing world to take policy steps to curb emissions. Other reasons for focusing on the BRICS and not including in the panel the US or the EU-28 countries (which are also large emitters) are as follows. First, negotiation of pollution abatement policies and emission targets are made on the basis of being part of certain groups. For instance, the Kyoto Protocol distinguished Annex I countries—corresponding mostly to rich countries—with formal emissions abatement obligations from non-Annex I countries—corresponding mostly to emerging and developing countries—with only voluntary commitments.^17^ Second, emissions projections are made taking into account the level of development of countries, for which it is easier to make assumptions about income growth, energy use and emissions patterns. Third, countries’ technological level is closely associated with the level of development, with richer countries having more advanced technology to save energy and curb emissions (Heil and Wodom 1999).

Fourth, the energy transition of the rich implies a shift from manufacturing to services, which brings a decline in energy use and GHG emissions. However, in emerging economies (that expand faster) and developing countries the transition is from an agrarian to an industrial economy, thus requiring higher energy use and emissions (Kander and Stern 2014). Fifth, according to the environmental Kuznets curve, high-income countries will reach the steady-state earlier than emerging and developing countries—that are expanding both their economies and their GHG emissions. Thus, rich countries achieve earlier lower emissions through reduced energy intensity and improved energy efficiency (Rajbhandari and Zhang 2018). According to the previous points, rich countries are expected to exhibit different convergence patterns from emerging and developing economies. In addition, income groups are expected to exhibit greater within-group convergence (Cesereklyei and Stern 2015).

On top of these, industrialized countries are far ahead in the fight against climate change than the BRICS. For instance, the EU pioneered the system for GHG emission allowance trading within the EU based on a “cap and trade principle” through Directive 2003/87/EC, subsequent amendments and its 2018 consolidation. Other examples are the Energy Efficiency Directive (2012/27/EC) and Renewable Energy Directive (2009/28/EC)—with their respective subsequent amendments—aimed at raising energy efficiency and the energy shift to renewables. Besides, the National Emissions Ceilings Directive (2001/81/EC) attempts to curb emissions by targeting emission reductions in NO_x_, NH_3_, NMVOC, SO_2_, and fine particulate matter.

As pointed out by Hoesly et al. (2018), GHG emissions before 1850 were driven by the burning of residential and agricultural biomass. With the onset of the industrial revolution, emissions associated with the industrial, energy and transportation sectors grew quickly, particularly in the mid-twentieth century. It was only at the end of the twentieth century that global emissions in some of the pollutants fell after the implementation of emission controls. However, rapidly growing economic activity in some of the BRICS in recent years led again to the increase of global emissions in some of the pollutants. We provide details in the next subsection.

### Evolution and current state of pollutants’ emissions

**Fig. 1:**
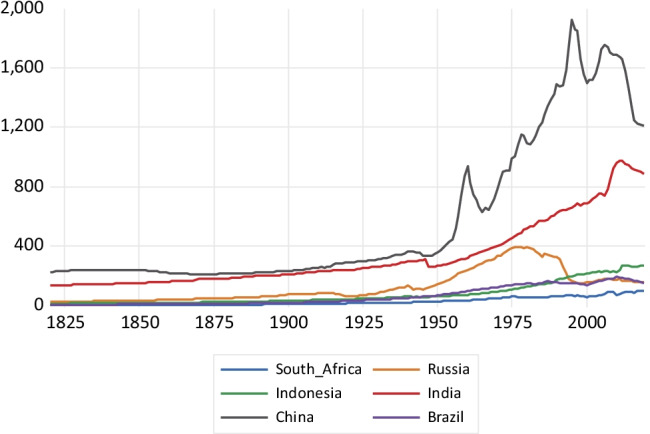
Black carbon emissions in kilotons

**Fig. 2:**
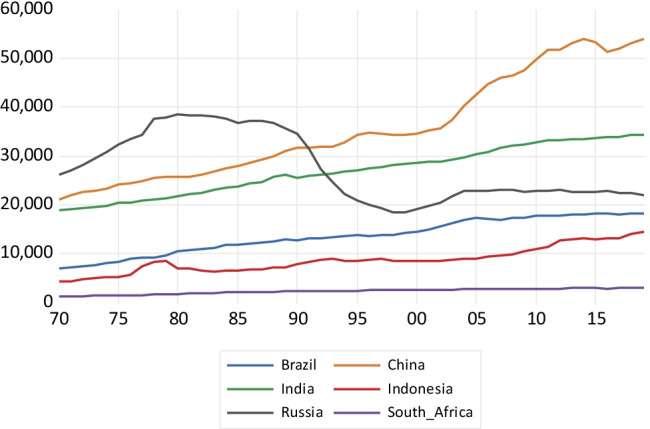
Methane emissions in kilotons

As shown in Fig. 1, a fast expansion in BC emissions takes place in China until 1995, reaching about 1800 kt of BC emissions and then considerably falling after 2005. The emissions growth in India is much less steep, reaching a value of about 900 kt around 2010 and falling afterwards. In the case of Russia, there is a fall since the late 1980s coinciding with the fall of the former Soviet Union. Indonesia, South Africa, and Brazil show a much more stable emissions profile, with a very slow emissions growth. Concerning CH_4_ emissions depicted in Fig. 2, China exhibits high growth, particularly after 2003, reaching about 53,000 kt of methane emissions. India, Indonesia, and Brazil exhibit a less steep emissions profile over the 1970–2018 period. Russia presents a sharp fall in methane emissions between 1990 and 1998, keeping a flat emissions profile afterwards.

**Fig. 3:**
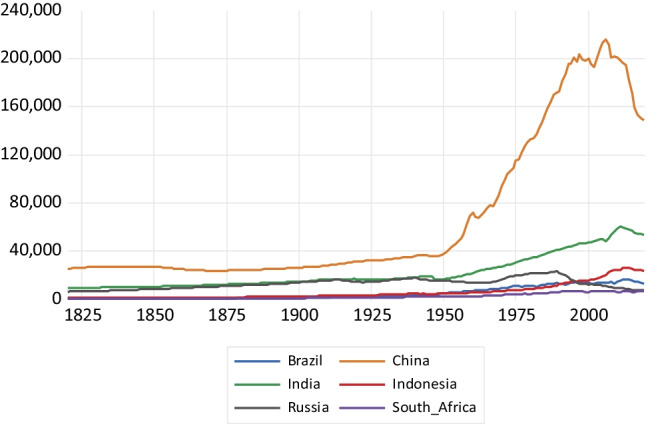
Carbon monoxide emissions in kilotons

**Fig. 4:**
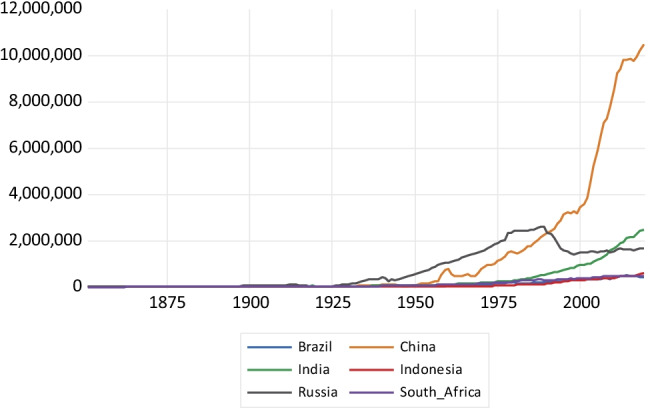
Carbon dioxide emissions in kilotons

As regards CO emissions presented in Fig. 3, China’s emissions take off after 1950 until about 2005, reaching around 215,000 kt and then experiencing an emissions reversal. India presents a less steep emissions profile, with a fall in emissions during the second decade of the twenty-first century. Russia shows a fall in emissions after the collapse of the Soviet Union, while the remaining countries exhibit a relatively flat profile. Regarding CO_2_ emissions drawn in Fig. 4, China presents a highly steep emissions profile surpassing 10 million kt of CO_2_ emissions by the end of the period. This is followed by India that exhibits a less steep profile. Again, Russia presents a reduction in CO_2_ emissions resulting from the economic decline associated with the Soviet Union collapse. The rest of the countries present a relatively flat emissions profile.

**Fig. 5:**
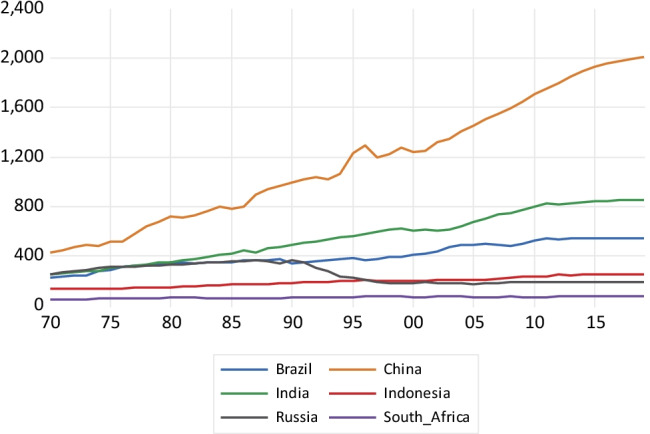
Nitrous oxide emissions in kilotons

**Fig. 6:**
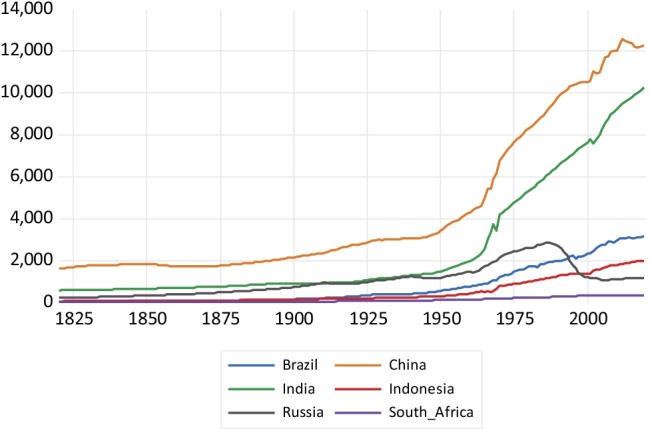
Ammonia emissions in kilotons

Concerning N_2_O emissions depicted in Fig. 5, China is again the country with the fastest growth over the period 1970–2018, reaching 2000 kt in 2018. This appears to be followed by India and Brazil that exhibit a less steep profile. Indonesia and South Africa present a fairly flat emissions profile. Russia again reduced emissions following the fall of the Soviet Union. In the case of ammonia emissions graphed in Fig. 6, both China and India present a very steep emissions profile, while Brazil and Indonesia exhibit much slower growth over the past five decades. South Africa’s emissions profile is fairly flat over the whole period. Again, Russia’ emissions decline after the economic depression associated with the fall of the Soviet Union.

**Fig. 7:**
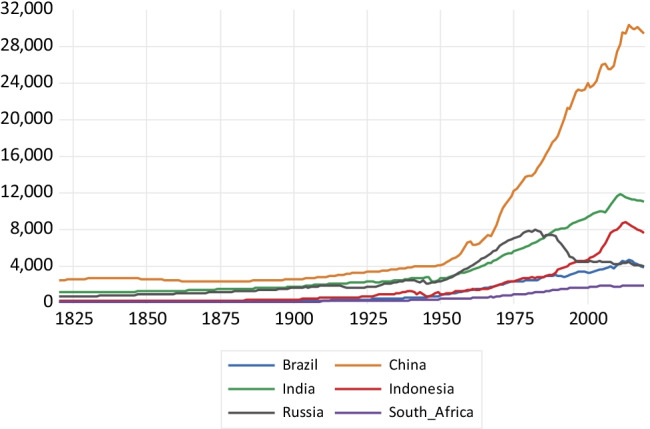
NMVOC emissions in kilotons

**Fig. 8:**
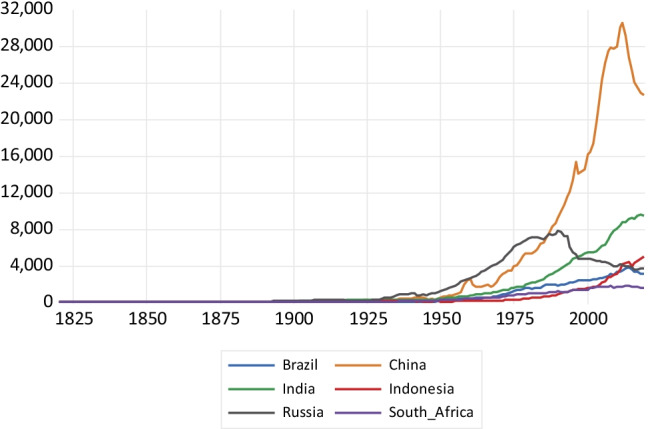
Nitrogen oxide emissions in kilotons

As far as NMVOC emissions—depicted in Fig. 7—are concerned, China exhibits a very steep profile since 1950, reaching more than 30,000 kt of NMVOC emissions around 2015 and only presenting a reversal of the trend around 2010. India and Indonesia present a slower expansion in NMVOC emissions since 1950, with a trend reversal around 2015. The emission profiles of Brazil and South Africa exhibit even a lower slope. Russian emissions decline after the economic collapse of the former Soviet Union. Similar patterns are observed for NO_x_ emissions drawn in Fig. 8. China presents a highly steep emissions profile, surpassing the 30,000 kt in 2011 and then observing a sharp decline afterwards. India and Indonesia exhibit a considerably less steep emissions profile, and Brazil and South Africa present much slower growth. Russia again shows a reversal of the trend in emissions caused by the economic collapse which followed the fall of the Soviet Union.

**Fig. 9:**
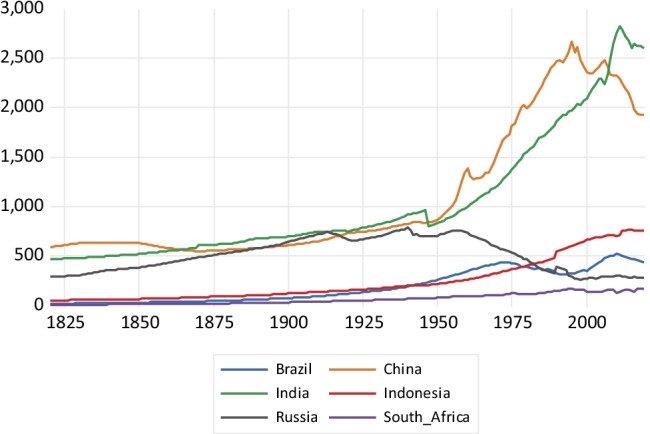
Organic carbon emissions in kilotons

**Fig. 10:**
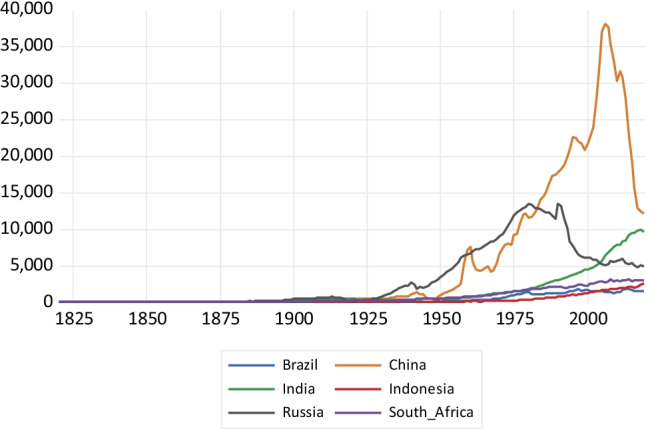
Sulfur dioxide emissions in kilotons

Concerning OC emissions drawn in Fig. 9, India is ahead of China due to widespread burning of biomass and coal for cooking and heating in extensive rural areas. Only around 2010, India’s emissions appear to fall. In China, the trend reversal is observed around 1995. Indonesia presents a much less steep emissions profile, while South Africa’s emissions profile is almost flat. Brazil exhibits a timid increase that reverses around 2010. Russian emissions again fall with the economic decline stemming from the collapse of the Soviet Union. Finally, Fig. 10 depicts SO_2_ emissions. China shows a sharp increase in SO_2_ emissions since 1950 to around 2005, reaching 38,000 kt of SO_2_ emissions. The trend appears to reverse sharply afterwards. India exhibits a less steep upward trend over the 1950–2017 period, whereas the emissions profiles of Brazil, Indonesia, and South Africa are much flatter. Russia exhibits again a reduction of SO_2_ emissions after the collapse of the Soviet Union, in line with Hoesly et al. (2018) who indicated that SO_2_ emissions are the most sensitive compound to economic activity changes.

In sum, we observe a specific pattern for the countries across all or most of the pollutants. China appears to be the country with the steepest emissions profile, though there is a trend reversal over the past decade coinciding with more stringent pollution control measures in power plants and transportation sectors, among others. India shows a less steep profile associated with slower emissions growth, though the trend reversal is more recent, if any, and less sharp. The emission profiles of Brazil, Indonesia, and South Africa are flatter, whereas Russia exhibits a sharp decline in emissions as a result of the collapse of the Soviet Union.

### Empirical strategy

Concerning the empirical strategy, this paper follows the work by Li and Papell (1999), but for the case of deterministic convergence of per capita pollutants emissions among the six BRICS countries. For that purpose, we compute the logarithm of the ratio of the per capita emissions series of the BRICS countries relative to the average per capita emission levels of the specific pollutant for a sample of 23 OECD countries.^18^ The variable for unit root testing is relative emissions, i.e., $R{E}_{it}=\ln \left(C{O}_{2_{it}}/{\overline{CO}}_{2_t}\right)$, where ${CO}_{2_{it}}$ relates to per capita CO_2_ emissions of the BRICS countries and ${\overline{CO}}_{2_t}$ is the yearly average per capita CO_2_ emission level for the 23 developed countries. *i=1,…,N* stands for the number of countries and *t* = 1,…,*T* for the time periods. We compute it this way because we aim at examining whether BRICS emissions converge to the emissions level of developed countries. Relative emissions are computed accordingly for the other nine emission series.

The definition of deterministic convergence implies mean stationarity in the log of relative emissions, thus requiring eliminating both deterministic and stochastic trends. Hence, emissions in the BRICS would move in parallel (i.e., converge deterministically) over the long-run relative to average OECD emissions.^19^

## Empirical results

In Figures (A1) to (A10) in the unpublished appendix, we depict the log of relative per capita emissions for the ten pollutants. With the exception of BC, OC, CO, and CO_2_ per capita emissions for which apparently there is a gradual narrowing of cross-country differences in per capita emissions over the long run, the graphical inspection does not indicate the existence of converging dynamics for CH_4_, N_2_O, NH_3_, NMVOC, NO_x_, and SO_2_ per capita emissions.

We formally examine the existence of pollutants emissions convergence via the linear panel unit root test of Chang (2004), the state-dependent nonlinear panel unit root tests of UO and EO, and the time-dependent nonlinear panel tests of OHS and OSS. Table 1 contains the panel statistics and the associated bootstrap p-values using the Sieve bootstrap methodology that control for cross-dependencies of unknown form. We will be able to infer which model best characterizes the converging dynamics of each of the pollutants once all tests are presented.

**Table 1:** Nonlinear panel unit root tests: baseline findings

	Linear	State-dependent nonlinear		Structural break	
	Chang	UO (ESTAR)	EO (AESTAR)	OHS (LSTR)	OSS (Fourier)
	${\overline{t}}_C$	${\overline{t}}_{UO}$	${\overline{F}}_{AE}$	${\overline{t}}_{\alpha }$	${\overline{t}}_{FIPS}$
BC	− 0.448 (0.955)	− 0.126 (0.930)	2.441 (0.478)	− 1.139 (0.732)	− 1.710 (0.863)
CH_4_	− 1.029 (0.971)	− 0.852 (0.925)	1.639 (0.852)	− 2.746** (0.010)	− 1.936 (0.907)
CO	1.712 (0.999)	1.137 (0.999)	12.603*** (0.000)	− 1.989 (0.901)	− 0.043 (0.999)
CO_2_	− 2.323** (0.034)	− 2.740** (0.030)	5.238** (0.033)	− 2.500 (0.235)	− 1.973 (0.518)
N_2_O	− 0.833 (0.953)	− 0.966 (0.971)	1.420 (0.922)	− 2.335 (0.185)	− 1.879 (0.874)
NH_3_	− 0.685 (0.976)	− 0.835 (0.961)	0.895 (0.996)	− 1.950 (0.941)	− 1.317 (0.989)
NMVOC	0.341 (0.999)	0.025 (0.999)	3.157 (0.194)	− 0.858 (0.999)	− 0.579 (0.998)
NO_x_	0.084 (0.999)	0.363 (0.999)	1.743 (0.842)	− 2.458* (0.095)	− 0.832 (0.995)
OC	− 0.174 (0.985)	− 0.568 (0.992)	0.936 (0.975)	− 2.254 (0.771)	− 1.188 (0.952)
SO_2_	0.644 (0.999)	0.792 (0.999)	3.636 (0.209)	− 1.945 (0.945)	− 0.276 (0.999)

***, **, and * imply rejection of the unit root null at the 1, 5, and 10% significance level

Column 1 in Table 1 presents the linear Chang (2004) test. It is remarkable that the joint nonstationarity null is only rejected for CO_2_ emissions at the 5% significance level. For the other nine pollutants, the evidence supports divergence of BRICS and Indonesia’ emissions to OECD levels. Since failure to reject the unit root null with linear tests can be due to the low power in the presence of nonlinearities, we next apply four panel unit root tests allowing for different nonlinear dynamics. Column 2 reports the UO test based on symmetric ESTAR adjustment dynamics. Again, the joint unit root null is only rejected for CO_2_ at the 5% level. Column 3 presents the evidence from the more flexible EO panel statistic allowing for asymmetric ESTAR dynamics. The joint unit root null is rejected for CO emissions at the 1% level and for CO_2_ at the 5% level. Columns 4 and 5 report the time-dependent nonlinear tests of OHS and OSS, respectively. They allow for a permanent structural break modelled by an LSTR function, and for multiple smooth breaks through the flexible Fourier function, respectively. It is remarkable that the OHS test rejects the nonstationarity null only for CH_4_ at the 5% level and NO_x_ emissions at the 10% level.

Table 2 presents the summary of results across all tests. These are the general identification rules to establish which specific model better captures the DGP of the converging dynamics for each pollutant. First, if the series rejects the unit root test with the linear test, the convergence process is considered linear stationary irrespective of other tests.^20^ This is the case of per capita CO_2_ emissions, which supports the prevalent finding in this literature favoring (linear) convergence in CO_2_ emissions, particularly among industrialized countries (Strazicich and List 2003; Westerlund and Basher 2008; Romero-Avila 2008; Chang and Lee 2008; Lee and Chang 2009; Awaworyi-Churchill et al. 2018).

**Table 2:** Summary table

	Linear	State-dependent nonlinear		Structural break		
	Chang	UO (ESTAR)	EO (AESTAR)	OHS (LSTR)	OSS (Fourier)	DGP
BC						I(1)
CH_4_				**+**		LSTR
CO			**+**			AESTAR
CO_2_	**+**	**+**	**+**			Linear
N_2_O						I(1)
NH_3_						I(1)
NMVOC						I(1)
NO_x_				**+**		LSTR
OC						I(1)
SO_2_						I(1)

+ indicates that the country-group rejects the null of nonconvergence for each specific panel unit root test

Second, if the series is stationary only by state-dependent tests, a state-dependent structure in the DGP prevails. The AESTAR test nests the ESTAR test. If both tests render stationarity, the process is symmetrical ESTAR. If the ESTAR test does not reject the unit root null, but the AESTAR test does, then the process is asymmetrical state-dependent.^21^ We find no single series for which the nonstationarity null is rejected with both the UO and EO tests, which would support the symmetric ESTAR model.^22^ However, the EO test rejects the unit root null for the CO series, whereas the UO test does not. Hence, the converging dynamics of CO emissions exhibit an AESTAR process. Third, if the series renders stationarity only in structural break tests, then the nonlinear structure of structural break form prevails. Our evidence indicates that CH_4_ and NO_x_ incorporate the single permanent structural break in their converging dynamics. Concerning BC, N_2_O, NH_3_, NMVOC, OC, and SO_2_ per capita emissions, the evidence clearly favors divergence since no single test rejects the joint nonstationarity null of no emissions convergence of the BRICS and Indonesia relative to the developed world average.

### Robustness check: convergence to Sweden’s emission levels

Arguably, the above exercise investigating the convergence of the BRICS and Indonesia to OECD emission levels may not be sufficiently ambitious to achieve the 1.5–2 °C target set in the Paris Climate Agreement, since the USA alone contributes by 15% to global emissions. Hence, as a robustness exercise, we measure convergence with respect to Sweden, which has absolute decoupling of pollution from economic activity.^23^^,^^24^ As a result, the finding of emissions convergence of the BRICS and Indonesia to the emission level of Sweden may be more supportive of the global emissions abatement agenda given by the 2030 Agenda for Sustainable Development and the Paris Agreement.

Remarkably, as reported in Table 3, the results are broadly in line with the baseline findings. N_2_O, NH_3_, OC, and SO_2_ emissions continue to exhibit divergence, whereas CH_4_, CO, and CO_2_ appear to converge deterministically to Sweden’s emission levels. This finding is again encouraging since CO_2_ and CH_4_ are the first and second main contributors to global emissions. The only major difference is that NMVOC shifts from divergence to AESTAR convergence, whereas NO_x_ shifts from LSTR converging dynamics to divergence. In addition, BC emissions shift from divergence to mixed evidence regarding the exact DGP behind deterministic convergence.

**Table 3:** Nonlinear panel unit root tests: relative emissions to Sweden

	Linear	State-dependent nonlinear		Structural break	
	Chang	UO (ESTAR)	EO (AESTAR)	OHS (LSTR)	OSS (Fourier)
	${\overline{t}}_C$	${\overline{t}}_{UO}$	${\overline{F}}_{AE}$	${\overline{t}}_{\alpha }$	${\overline{t}}_{FIPS}$
BC	− 1.966 (0.329)	− 7.659 *** (0.000)	59.736*** (0.000)	− 2.127 (0.732)	− 3.646* (0.079)
CH_4_	− 0.198 (0.991)	0.008 (0.999)	1.662 (0.998)	− 2.676** (0.016)	− 1.110 (0.983)
CO	− 2.323** (0.034)	− 0.532 (0.999)	4.535 (0.134)	− 1.095 (0.999)	0.191 ( 0.998)
CO_2_	− 2.043 (0.112)	− 2.958** (0.032)	7.904** (0.025)	− 3.086 (0.101)	− 1.938 (0.795)
N_2_O	− 0.685 (0.943)	− 0.561 (0.940)	0.803 (0.978)	− 2.373 (0.932)	− 2.395 (0.502)
NH_3_	− 0.512 (0.963)	− 1.218 (0.749)	3.569 (0.145)	− 0.849 (0.999)	− 0.696 (0.993)
NMVOC	− 0.632 (0.849)	− 2.417 (0.152)	7.992** (0.034)	− 1.051 (0.999)	− 0.938 (0.966)
NO_x_	− 0.924 (0.868)	− 0.829 (0.902)	4.266 (0.163)	− 2.294 (0.850)	− 2.026 (0.720)
OC	− 0.778 (0.858)	− 2.244 (0.232)	4.641 (0.112)	− 0.656 (0.999)	− 1.893 (0.756)
SO_2_	0.329 (0.998)	0.732 (0.999)	2.286 (0.576)	− 0.667 (0.999)	− 1.893 (0.992)

***, **, and * imply rejection of the unit root null at the 1, 5, and 10% significance level

## Conclusion and policy implications

The above results contain important policy implications at the technical level. For carbon dioxide emissions, convergence appears to occur linearly, which supports the continuation of current abatement policies to curb carbon dioxide emissions. However, the finding of asymmetric size nonlinearity in carbon monoxide emissions implies that environmental policy will react more aggressively in the event of large deviations from the emissions target. Besides, environmental authorities have also the possibility to speed up the convergence process when emissions are above the target by virtue of the AESTAR process. Concerning methane and nitrogen oxides emisions that exhibit time-dependent nonlinearity, environmental authorities can curb emissions by controlling this long-term smooth stationary upward-trend structure and even reverse its dynamics.

The results are partly encouraging since for four of the pollutants (including the main greenhouse gas, carbon dioxide, in addition to methane—the second main contributor to global emissions—carbon monoxide and nitrogen oxides), emission convergence of the BRICS (that comprise major emitters like China or India) and Indonesia will encourage other emerging economies and the developing world to take steps to curb their emissions. This is key to achieve the full 2030 Agenda for Sustainable Development (particularly Sustainable Development Goal 13 on the control of climate change globally) and the Paris Agreement on climate change. The achievement of the 2030 Agenda will involve the improvement of human capital and institutional capacity building on climate change mitigation, adaptation and planning, the strengthening of adaptive capacity to climate-related disasters and the mobilization of $100 billions annually to address climate change in the developing world.

Also relevant for other emerging and developing countries is the fact that convergence in these key compounds (CO_2_, CH_4_, CO, and NO_x_) supports the adoption of a per capita emissions allocation scheme without the need for substantial resource transfers in the international emissions market or cross-border movements of high-pollution plants. This is expected to save a large amount of resources for those economies achieving convergence. In addition, emissions convergence makes it easier for countries to harmonize anthropogenic emissions abatement legislation, in addition to being a key ingredient in emissions projection models guiding the formulation of climate change abatement policies. Besides, the BRICS, other emerging economies and developing countries should follow the footsteps of EU countries that adopted the National Emissions Ceilings Directive (2001/81/EC). This Directive tries to curb emissions by setting emission reduction targets on five main pollutants: NO_x_, NH_3_, NMVOC, SO_2_, and fine particulate matter.

In addition, even though the BRICS have not pledged to contribute to the Green Climate Fund that seeks to mobilize resources for adaptation and mitigation actions in developing countries, the BRICS have created the New Development Bank^25^ to channel funds for infrastructure and sustainable development in the BRICS and other developing countries (Downie and Williams 2018). They also created the Contingent Reserve Arrangement and the Asian Infrastructure Investment Bank as further mechanisms to finance green projects (Petrone 2019). In addition, Brazil, India, and China have made extensive use of the Clean Development Mechanism (initially created under the Kyoto Protocol) to finance clean technologies and infrastructures.

As for the rest of pollutants, BRICS and Indonesian emission levels appear to diverge from those of the OECD. Failure to converge makes it more difficult to harmonize internationally greenhouse gas emissions abatement legislation. This indicates that the BRICS and Indonesia should develop policy programmes to curb their emissions of black carbon, nitrous oxide, ammonia, non-methane volatile organic compounds, organic carbon and sulfur dioxide. More specifically, the BRICS and Indonesia should follow the footsteps of the developed world and implement ambitious programmes to curb emissions. First, sulfur dioxide emissions should be reduced by the installation of flue gas desulfuration on electric power plants, progressive measures to remove sulfur from the combustion of crude oil and coal, and the prohibition of bunker fuel with high sulfur content in transoceanic shipping (Smith et al. 2011).^26^ Second, nitrous oxide emissions can be controlled by reducing synthetic nitrogen-based fertilizer applications in agriculture, reducing fossil fuel consumption in motor vehicles and controlling their pollution via catalytic converters, as well as technological upgrading and fuel switching to curb emissions from fossil fuel combustion at industry (U.S. Environmental Protection Agency 2021). Along similar lines, ammonia emissions can be curbed by controlling synthetic fertilizer application and manure management in agriculture.

Third, there should be more stringent non-methane volatile organic compounds emission standards in the energy transformation and extraction sectors as well as in the manufacturing of paints and solvents, in the same way catalytic converters have considerably reduced transport non-methane volatile organic compounds emissions. Fourth, the control of organic carbon emissions—and to some extent black carbon emissions—is more difficult since they mainly stem from residential sector cooking and heating using biomass by expanding rural populations. Alternative products should be favored over products of incomplete combustion. Limiting the expansion of diesel vehicles and coke production in the energy transformation sector can also reduce black carbon emissions.

Other facts can favor the future convergence of BRICS and Indonesian emissions to advanced countries levels. As an initiative to achieve the full decarbonization of economies by 2050, the European Commission has recently proposed a new carbon border adjustment mechanism, which sets a carbon price on imports of products with a high-pollution capacity such as cement, iron, steel, aluminium, oil, chemical products and nitrogen-based fertilizers. The aim of this policy measure is to make countries outside Europe with carbon-intensive production (particularly countries such as China and India) embark actively on the climate change combat.^27^ Furthermore, BRICS Environment Ministers should hold more meetings to continue the collective global efforts against climate change guided by equity, national priorities and circumstances, and the principles of “Common but Differentiated Responsibilities and Respective Capabilities,” as negotiated in the Paris Agreement. This includes cooperation on renewable energy sources and green technologies, sustainable cities and habitats, clean rivers, creation of carbon sinks (forestry), biodiversity conservation, waste management, and prevention of air and water pollution, among others. To achieve this, the BRICS urge developed countries to mobilize financial resources by $100 billions per year (which is the goal for climate finance) as well as provide technological and capacity building to improve their capacity to abate emissions.

The different interests of the BASIC coalition (with India and particularly China being large fossil fuel importers) and a large fossil fuel exporter like Russia may be partly responsible for the lack of emissions convergence of the BRICS group to advanced countries levels for six of the pollutants studied. Hence, more efforts should be made for the BRICS to align their economic, energy and climate interests to be able to act as a coherent bloc, which would help them converge to rich countries’ emission levels.

A limitation of this study is the failure of the authors to employ nonlinear hybrid panel unit root tests that combine state-dependence of the ESTAR or AESTAR type and time-dependence in the form of structural breaks. Hence, a future avenue of research is to develop hybrid panel unit root statistics, which will extend the univariate nonlinear hybrid unit root tests of Christopoulos and Leon-Ledesma (2010), Omay and Yildirim (2014), and Omay et al. (2018a). These combine symmetric and asymmetric ESTAR adjustment with a structural-break functional form. Once developed, we aim at applying them to panels of emissions disaggregated at several levels: (1) regional or state level along the lines of Burnett (2016), (2) sectoral level along the lines of Yu et al. (2018), and (3) both regional and sector levels following the work of Bolea et al. (2020).

## Supplementary information

ESM 1 (DOCX 511 kb)

ESM 2 (ZIP 232 kb)

ESM 3 (ZIP 235 kb)

ESM 4 (DOCX 11.9 kb)

## Data Availability

The link to the RATS codes for the computation of the nonlinear panel statistics along with the datasets are available in the Journal’s homepage. Please cite this article when employing these codes. The data on pollutant emissions are freely available at http://www.globalchange.umd.edu/CEDS/ and population figures at https://www.rug.nl/ggdc/historicaldevelopment/maddison/. Supplementary materials are available online in the Journal’s homepage.

## Footnotes

1 Global sulfur emissions flatten after 2005, since falling emissions in Europe, North America, and Russia are offset by rising emissions in China and other parts of Asia. It is remarkable that China’s emissions began to decrease in the energy transformation sector after the introduction of emission controls in power plants in 2005, though emissions in industrial processes remain uncontrolled, thus becoming dominant nowadays (Hoesly et al. 2018)

2 Non-methane volatile organic compounds is another important source of ground-level toxic gas ozone, which is a GHG contributing to global warming.

3 According to Hoesly et al. (2018), global nitrogen oxides emissions flatten around 2008 due to the fact that the rise in industrial emissions after 2000 is cancelled out by the fall in international shipping emissions. The introduction of stringent pollution controls in power plants and catalytic converters in the transportation sector can explain the decline in emissions in North America, Europe, and more recently China and other parts of Asia.

4 Global methane emissions increased substantially by 36% over the past three decades. Despite the fall in North America and Europe, carbon dioxide emissions continued to steadily rise over the past decades, due to the steady increase in China and Asia as well as the moderate rises in Africa and Latin America (Hoesly et al. 2018).

5 Acosta-Navarro et al. (2017) calculated the cooling effect, finding that a decline in aerosol emissions from fossil fuels under a maximum technically feasible reduction scenario causes a global and Arctic warming of 0.26 to 0.84 K, respectively. By contrast, under the representative concentration pathway 4.5 emission scenario, fossil fuel emissions causing the GHG effect raise global and Arctic average surface termperature by 0.35 and 0.94 K, respectively.

6 This article provides an extremely condensed summary of the environmental policy measures implemented in each country, so that the reader has some background on this issue. However, due to space limitations, it is beyond the scope of this paper to provide a detailed account of all policy measures implemented in each country to curb emissions. For that purpose, we refer the reader to the Climate Action Tracker available at https://climateactiontracker.org/.

7 Though signing the Paris Agreement in April 2016, Russia is the only BRICS member that has not ratified the Agreement yet.

8 This INDC has been replaced by the 2030 new emissions target in November 2020, setting the limit to a 30% reduction in emissions below 1990 levels. Thus, this does not constitute any real advancement.

9 As pointed out by Zheng et al. (2019), China is the largest consumer of fired-coal in power generation and also the largest producer of solar technology. The achievement of the Paris Agreement target largely depends on whether this and other green technologies crowd out old highly inefficient and polluting technologies like coal firing.

10 Strictly speaking, the BRICS group does not include Indonesia. Nevertheless, this emerging country shares many economic features of the BRICS. It also has a large population of almost 300 million inhabitants. Given the similarities, we have decided to include this country in the BRICS group for analytical purposes.

11 The EU Renewable Energy Directive imposes further restrictions so that palm-oil plantations are sustainable and do not drive deforestation.

12 Biofuel production consists of mainly biodiesel, but the government intends to promote the production of bioethanol.

13 See Pettersson et al. (2014) and Payne (2020) for thorough reviews of studies on emissions convergence, including work on conditional β-convergence, distributional dynamics or club convergence following Phillips and Sul (2007).

14 It is based on univariate LNV tests.

15 We compute per capita emission levels of the pollutants using long-term population data from the Maddison Project Database (2020).

16 According to the Intergovernmental Panel on Climate Change (2014), almost 76% of global total emissions would correspond to CO_2_ emissions, with 65% related to fossil fuel combustion and industrial processes and 11% related to deforestation and other land uses.

17 Apparently, this differentiation disappeared with the Paris Agreement, but in practice countries negotiate as groups, which are again associated with the level of development: EU-28 countries appear closely aligned with other rich OECD members, the BASIC group formed by four BRICS, or the G77 group of developing countries.

18 The OECD sample used to calculate the developed world average includes Australia, Austria, Belgium, Canada, Switzerland, Chile, Germany, Denmark, Spain, Finland, France, United Kingdom, Greece, Ireland, Italy, Japan, Netherlands, Norway, New Zealand, Portugal, Sweden, Turkey, and the USA.

19 This definition is preferred to stochastic convergence which allows for permanent differences in per capita emission levels across countries through the presence of a linear trend.

20 It is very likely that other nonlinear panel unit root tests confirm stationarity if the data are linear, because they conserve their statistical power in the case of linearity.

21 This occurs because if asymmetry is present, the ESTAR test is unable to detect stationarity.

22 The only series for which both UO and EO tests reject the unit root null is CO_2_, which is shown to be linear stationary.

23 We thank an anonymous referee for suggesting this exercise.

24 Jakob et al. (2012) find some evidence of decoupling of economic activity and energy use patterns among 30 developing countries and 21 industrialized countries over the period 1971–2005.

25 It was agreed in the sixth BRICS summit in July 2014.

26 Sulfur dioxide emissions in energy transformation in China fell since 2005 with the introduction of emissions controls in power plants. However, uncontrolled industrial emissions have become dominant nowadays (Hoesly et al. 2018).

27 See more details in https://greenfiscalpolicy.org/brics-summit-is-likely-to-strongly-oppose-carbon-tax-proposed-by-eu/, accessed in November 2021.
