# Supplementary material for: A long-run convergence analysis of aerosol precursors, reactive gases, and aerosols in the BRICS and Indonesia: is a global emissions abatement agenda supported?
Source: Environ Sci Pollut Res Int. 2022 Sep 29;30(6):15722–39. doi: 10.1007/s11356-022-22988-9 (PMC9908704; doi:10.1007/s11356-022-22988-9)
Supplement: Supplementary file 1 — (DOCX 511 kb) [file 11356_2022_22988_MOESM1_ESM.docx]

**A Long-run Convergence Analysis of Aerosol Precursors, Reactive Gases and Aerosols in the BRICS and Indonesia: Is a Global Emissions Abatement Agenda Supported?**

Diego Romero-Avila & Tolga Omay

*Pablo de Olavide University, Atılım University*

**UNPUBLISHED APPENDICES: NOT INTENDED FOR PUBLICATION**

**APPENDIX A: FIGURES**

**Figure A1: Evolution of Relative per Capita BC Emissions**

**Figure A2: Evolution of Relative per Capita CH_4_ Emissions**

**Figure A3: Evolution of Relative per Capita CO Emissions**

**Figure A4: Evolution of Relative per Capita N_2_O Emissions**

**Figure A5: Evolution of Relative per Capita NH_3_ Emissions**

**Figure A6: Evolution of Relative per Capita NMVOC Emissions**

**Figure A7: Evolution of Relative per Capita NO_x_ Emissions**

**Figure A8: Evolution of Relative per Capita OC Emissions**

**Figure A9: Evolution of Relative per Capita SO_2_ Emissions**

**Figure A10: Evolution of Relative per Capita CO_2_ Emissions**

**APPENDIX 2. ECONOMETRIC METHODOLOGY**

Having presented the literature review, we now shift the focus to provide a brief explanation of the newly proposed nonlinear unit root tests used in the empirical analysis. As pointed out above, UO and EO are classified as state-dependent nonlinear panel unit root tests, and OHS and OSS as structural break panel unit root tests.

**3.1 EO Test**

EO (2014) start with the following nonlinear asymmetric heterogeneous panel specifications:

| $\Delta y_{it}=G_{it}(\gamma_{1i},y_{i,t-1})\{S_{it}(\gamma_{2i},y_{i,t-1})\rho_{1i}+(1-S_{it}(\gamma_{2i},y_{i,t-1}))\rho_{2i}\}y_{i,t-1}+\varepsilon_{it},$ | (1) |
| --- | --- |
| $G_{it}(\gamma_{1i},y_{i,t-1})=1-\exp(-\gamma_{1i} y_{i,t-1}^{2}) \gamma_{1i}\geq0 \text{for} \text{all} i$ | (2) |
| $S_{it}(\gamma_{2i},y_{i,t-1})=[1+\exp(-\gamma_{2i} y_{i,t-1})]^{-1} \gamma_{2i}\geq0 \text{for} \text{all} i$ | (3) |

where $\varepsilon_{it}\sim iid(0,\sigma_{i}^{2})$. If $\gamma_{1i}>0$ and $\gamma_{2i}\to\infty$, the size of the deviation is large for the state variable ($y_{i,t-1}$) and an ESTAR transition occurs between the central regime and outer regime model, with $\gamma_{1i}$ determining the speed of the transition. If the deviation is in the negative direction of the state variable, the outer regime is $\Delta y_{it}=\rho_{i2}y_{i,t-1}+\varepsilon_{it}$, and if the deviation is in the positive direction the outer regime is $\Delta y_{it}=\rho_{i1}y_{i,t-1}+\varepsilon_{it}$, where the transition functions take the extreme values 0 and 1, respectively, for these two cases. If $\rho_{i1}\neq\rho_{i2}$ $\text{for} \text{all} i$, the autoregressive adjustment is asymmetric. Note that Eq. (1) nests the panel symmetric ESTAR specification of the UO test when $\rho_{1i}=\rho_{2i}=\rho_{i}$ $\text{for all }i$. Due to the extreme assumption $\gamma_{2i}\to\infty$, the logistic function reduces to a simple step function and behaves like the TAR model. Asymmetry can also occur for small and moderate values of $\gamma_{2i}$. Nevertheless, under the other extreme value for $\gamma_{2i}$ $(i.e.,\text{ }\gamma_{2i}\to0)$, irrespective of the values of $\rho_{1i}$ and $\rho_{2i},$ the composite function $G_{it}(\gamma_{1i},y_{i,t-1})\{S_{it}(\gamma_{2i},y_{i,t-1})\rho_{1i}+(1-S_{it}(\gamma_{2i},y_{i,t-1}))\rho_{2i}\}$ becomes symmetric because $S_{it}(\gamma_{2i},y_{i,t-1})\to0.5$ $\text{for} \forall t \text{and} \forall i$. As a result, this feature can be used to test whether the series under study has symmetric or asymmetric dynamics.

In case the errors in Eq. (1) are serially correlated, it can be extended to allow for higher order dynamics:

| $\Delta y_{it}=G_{it}\left(\gamma_{1i},y_{i,t-1}\right)\left\{S_{it}\left(\gamma_{2i},y_{i,t-1}\right)\rho_{1i}+\left(1-S_{it}\left(\gamma_{2i},y_{i,t-1}\right)\right)\rho_{2i}\right\}y_{i,t-1}+\sum_{j=1}^{p_{i}} \delta_{ij}\Delta y_{i,t-j}+\varepsilon_{it}$ | (4) |
| --- | --- |

The unit root hypothesis can be tested against the alternative hypothesis of globally stationary symmetric or asymmetric ESTAR nonlinearity with a unit root central regime by testing $H_{0}:\gamma_{1i}=0$ in Eq. (1). However, there are unidentified parameters under this null, such as $\gamma_{2i}$, $\rho_{1i}$ and $\rho_{2i}$. In order to solve the unidentified parameters problem, the composite function must contain two different transition functions and, thus, a Taylor approximation both around $\gamma_{1i}=0$ and $\gamma_{2i}=0$ should be employed. We follow Sollis (2009) and obtain the auxiliary equation in two steps within a panel framework. Replacing $G_{it}(\gamma_{1i},y_{i,t-1})$ in Eq. (1) with a first-order Taylor expansion around $\gamma_{1i}=0$ gives

| $\Delta y_{it}=\rho_{1i}\gamma_{1i}y_{i,t-1}^{3}S_{it}\left(\gamma_{2i},y_{i,t-1}\right)+\rho_{2i}\gamma_{1i}y_{i,t-1}^{3}\left(1-S_{it}\left(\gamma_{2i},y_{i,t-1}\right)\right)+\varepsilon_{it}$ | (5) |
| --- | --- |

Replacing $S_{it}(\gamma_{2i},y_{i,t-1})$ in Eq. (4) with a first-order Taylor expansion around $\gamma_{2i}=0$ yields

| $\Delta y_{it}=a(\rho_{2i}^{*}-\rho_{1i}^{*})\gamma_{1i}\gamma_{2i}y_{i,t-1}^{4}+\rho_{2i}^{*}\gamma_{1i}y_{i,t-1}^{3}+\varepsilon_{it}$ | (6) |
| --- | --- |

where $a=1/4$. Rearranging the coefficients as and [[1]](#footnote-1) we obtain the following auxiliary equation

|  | (7) |
| --- | --- |

Eq. (7) can be extended to render an augmented version

|  | (8) |
| --- | --- |

The null hypothesis $H_{0}:\gamma_{1i}=0$ for all $i$ in Eq. (1) becomes for all $i$ in the auxiliary model. The panel statistic is computed by taking the average of the individual $F_{i,AE}$statistics as follows:

| $\bar{F}_{AE}=N^{-1}\sum_{i=1}^{N} F_{i,AE}.$ | (9) |
| --- | --- |

Sollis (2009) stated that individual $F_{i,AE}$ statistics have a non-standard $F-$distribution. This is carried over to the panel $\bar{F}_{AE}$ test statistic that has also a non-standard distribution and exact critical values of $\bar{F}_{AE}$ can be computed via stochastic simulation for different values of *N* and *T*. In addition, if the unit root hypothesis ( for all $i$) is rejected, then the null hypothesis of symmetric ESTAR nonlinearity can be tested against the alternative of asymmetric ESTAR nonlinearity such that for all $i$ against in Eq. (8). Under the symmetric null hypothesis, Sollis (2009) employed the individual $t-$statistic ($t_{i,AE}^{as}$) with a standard $t-$distribution. In the panel framework, $\bar{t}_{AE}^{as}$ can be obtained as the average of individual statistics, which has a standard distribution.

The limiting distribution of these test statistics is valid under the assumption of *i.i.d.* disturbances. However, if the disturbances are not independent, the limit distributions of these statistics proposed here are no longer valid and are unknown in the presence of cross-correlations among the cross-section units. We cope with this problem by computing the empirical distributions of $\bar{F}_{AE}$ and $\bar{t}_{AE}^{as}$ test statistics using the Sieve bootstrap methodology, pioneered by the work of Chang (2004).

**3.2 OHS Test**

OHS (2018) develop a structural break panel unit root test, with
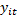
 being generated as follows:

| $y_{it}=\phi_{it}+x_{it}$ | (10) |
| --- | --- |
| $x_{it}=\mu_{i}x_{it-1}+\varepsilon_{it}$ | (11a) |

for $i=1....,N$cross-section units and $t=1....,T$ time periods. Here, $\phi_{it}$ represents the (slow moving) deterministic trend function. Initially, we assume that the errors, $\varepsilon_{it}$ are zero mean processes distributed independently across both $i$ and
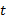
, that is,
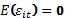
, $E\left(\varepsilon_{it}^{2}\right)=\sigma^{2}$and cross-sectionally and serially uncorrelated,
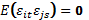
 for $i\neq j$and$t\neq s$. Consider the null hypothesis of a unit root, $\mu_{i}=1$, for all $i$ in Eq. (11a), in which case it can be rewritten as

| $\Delta x_{it}=\rho_{i}x_{it-1}+\varepsilon_{it}$ | (11b) |
| --- | --- |

where $\rho_{i}=-\left( 1-\mu_{i} \right).$ Now, the null hypothesis of a unit root becomes: $H_{0}:\rho_{i}=0$ for all $i$, while the alternative hypothesis of stationarity $H_{1}:\rho_{i}<0$ for some $i$. Note that the component representation, (10), enables us to maintain the same deterministic trend function both under the null and alternative hypotheses (Schmidt and Phillips, 1992). The OHS test models the smooth transition function,
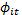
, by the logistic one:

| Model A 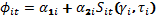 | (12a) |
| --- | --- |
| Model B 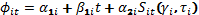 | (12b) |
| Model C 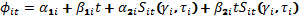 | (12c) |

where
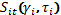
 are the individual-specific LSTAR functions:

| 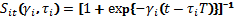, 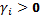 | (13) |
| --- | --- |

for
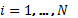
and *t* = 1,…,*T*. The transition function
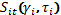
 is continuous, bounded between zero and one, and controls the transition from one regime to another. The parameter $\tau_{i}$ entails the timing of the transition midpoint as one obtains
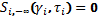
,
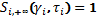
, and
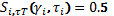
 for
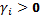
. The parameter
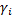
 implies the smoothness of transition. For small values of
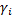
,
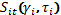
 crosses the interval
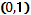
 very slowly, and
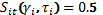
 for all values of
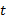
 in the limiting case $\gamma_{i}=0$. For large values of $\gamma_{i}$,
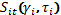
changes from 0 to 1 instantaneously at time
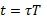
. Therefore, the logistic transition function in (13) nests the no-break and the instantaneous break model as a special case. In particular, if
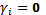
, then
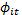
 reduces to a constant in (12a) or to a constant and linear trend in (12b) and (12c). In addition, as $\gamma_{i}$ tends to infinity, the model allows for an instantaneous break at time
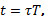
 (Perron, 1989). If $x_{it}$ is a mean-zero $I(0)$ process, then $y_{it}$ in (12a) is stationary around the mean that changes from an initial value, $\alpha_{1i}$ to the final value,
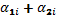
 under the identifying condition,
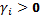
. Likewise, the process in (12b) is stationary around changing means but fixed slopes of trends, while the process in (12c) is stationary around changing means and gradually changing trend slopes from $\beta_{1i}$ to
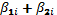
.

**3.3. OSS Test**

OSS (2021) develop a multiple structural break panel unit root test by allowing $y_{i,t}$ to be the panel flexible Fourier function form on the time domain $t=1,2,...,T$ for the cross section units $i=1,2,...,\mathrm{N}$. Assume $y_{i,t}$ follows the data generating process (DGP) with heterogeneous intercept parameter$\alpha_{i}$:

|  | (14) |
| --- | --- |

where $\varepsilon_{i,t}$ is a stationary disturbance with variance $\sigma_{i}^{2}$. The initial value is assumed to be fixed, and $\varepsilon_{i,t}$ to be weakly dependent as in EL. If the functional form of $d_{i}(t)$ is known, Eq. (14) can be estimated and the null hypothesis of joint nonstationarity be tested. However, when the form of $d_{i}(t)$is unknown, any test for is difficult to implement if $d_{i}(t)$is miss-identified. The OSS test is based on approximating $d_{i}(t)$through the Fourier expansion as follows:

|  | (15) |
| --- | --- |

where $k_{i}$ indicates a particular fractional frequency and $T$is the number of observations. When no nonlinear trend is present, all values of $\beta_{1i}=\beta_{2i}=0$, which renders the IPS test as a special case. In addition, the homogenous frequency assumption $k_{i}=k=1$ yields:

| $\Delta y_{i,t}=\alpha_{i}+\rho_{i}y_{i,t-1}+d_{i}(t)+e_{i,t}$ | (16) |
| --- | --- |

The hypotheses for unit root testing based on Eq. (16) are as follows:

| $H_{0}:\rho_{i}=0,$ for all i (linear nonstationary) |  |
| --- | --- |
| $H_{0}:\rho_{i}<0,$ for some i (stationary around nonlinear trend) (17) |

OSS develop a panel unit root test computed as the average of individual $FIPS$statistics. The $FIPS$ statistic for the $i^{th}$-individual is simply the $t-ratio$ of $\rho_{i}$ in Eq. (16) defined by

| $t_{i,fr}(N,T)=\frac{\Delta y_{i}^{'}M_{z}y_{i,-1}}{\hat{\sigma}_{i,fr}\left(y_{i,-1}^{'}M_{z}y_{i,-1}\right)^{1/2}}$ | (18) |
| --- | --- |

where$\Delta y_{i}=\left(\Delta y_{i,1},\Delta y_{i,2},...,\Delta y_{i,\mathrm{T}}\right)^{'}$,$y_{i,-1}=\left(\Delta y_{i,0},\Delta y_{i,1},...,\Delta y_{i,\mathrm{T}-1}\right)^{'}$ $M_{z}=I_{T}-Z\left(Z^{'}Z\right)^{-1}Z^{'}$,$Z=\left(\tau,ϒ_{1},ϒ_{2}\right)$ $\tau=\left(1,1,...,1\right)^{'}$ $ϒ_{1}=\left(\sin\left(2\pi k1/\mathrm{T}\right),\sin\left(2\pi k2/\mathrm{T}\right),...,\sin\left(2\pi k\mathrm{T}/\mathrm{T}\right)\right)^{'}$ $ϒ_{2}=\left(\cos\left(2\pi k1/\mathrm{T}\right),\cos\left(2\pi k2/\mathrm{T}\right),...,\cos\left(2\pi k\mathrm{T}/\mathrm{T}\right)\right)^{'}$ and $\hat{\sigma}_{i,fr}$ is the consistent estimator such that $\hat{\sigma}_{i}^{2}=\Delta y_{i}^{'}M_{i,z}\Delta y_{i}/\left(T-4\right)$ in which $M_{i,z}=I_{T}-G_{i}\left(G_{i}^{'}G_{i}\right)^{-1}G_{i}^{'}$and$G_{i}=\left(Z,y_{i,-1}\right)$For a fixed $T$, the panel statistic is:

| $\bar{t}=\frac{1}{N}\sum_{i=1}^{N} t_{i}$ | (19) |
| --- | --- |

The UO panel unit root test can be explained on the basis of the EO test. More specifically, it is the symmetric version of the EO test, which is built as the average of individual KSS tests. All tests use the Sieve bootstrap algorithms explained extensively in the corresponding articles.

1. Our notation follows that in Sollis (2009). [↑](#footnote-ref-1)
