# Supplementary material for: A long-run convergence analysis of aerosol precursors, reactive gases, and aerosols in the BRICS and Indonesia: is a global emissions abatement agenda supported?
Source: Environ Sci Pollut Res Int. 2022 Sep 29;30(6):15722–39. doi: 10.1007/s11356-022-22988-9 (PMC9908704; doi:10.1007/s11356-022-22988-9)
Supplement: Supplementary file 4 — (DOCX 11.9 kb) [file 11356_2022_22988_MOESM4_ESM.docx]

**A long-run convergence analysis of aerosol precursors, reactive gases, and aerosols in the BRICS and Indonesia: Is a global emissions abatement agenda supported?**

Diego Romero-Ávila & Tolga Omay

The five statistics employed on this paper: Diego Romero-Ávila & Tolga Omay, 2022. A long-run convergence analysis of aerosol precursors, reactive gases, and aerosols in the BRICS and Indonesia: Is a global emissions abatement agenda supported? *Environmental Science and Pollution Research*, forthcoming, are computed with a **RATS library** that was created for the following publication:

Diego Romero-Ávila & Tolga Omay, 2022, Convergence of per capita energy consumption around the world: New evidence from nonlinear panel unit root tests, Energy Economics, Volume 111, 106062.

As such, they were made available when the article was published, and they can be obtained through the following link:

<https://www.sciencedirect.com/science/article/pii/S0140988322002286>

There, we provide details on how to use the RATS library.

If you use the nonlinear panel unit root tests RATS library, please cite the following two articles:

Diego Romero-Ávila, Tolga Omay, 2022, Convergence of per capita energy consumption around the world: New evidence from nonlinear panel unit root tests, Energy Economics, Volume 111, 106062.

Diego Romero-Ávila, Tolga Omay, 2022. A long-run convergence analysis of aerosol precursors, reactive gases, and aerosols in the BRICS and Indonesia: is a global emissions abatement agenda supported? Environmental Science and Pollution Research, forthcoming.
